# Cytokine Response to Exercise and Its Modulation

**DOI:** 10.3390/antiox7010017

**Published:** 2018-01-17

**Authors:** Katsuhiko Suzuki

**Affiliations:** Faculty of Sport Sciences, Waseda University, 2-579-15 Mikajima, Tokorozawa, Saitama 359-1192, Japan; katsu.suzu@waseda.jp; Tel.: +81-4-2947-6898

**Keywords:** cytokine, chemokine, neutrophil, macrophage, inflammation

## Abstract

Strenuous exercise induces such inflammatory responses as leukocytosis (neutrophilia) and symptoms as delayed-onset muscle soreness and swelling. However, the association between inflammatory mediator cytokines and oxidative stress is not fully delineated. Herein, in addition to basic background information on cytokines, research findings on exertional effects on cytokine release and the underlying mechanisms and triggers are introduced. Then, the associations among cytokine responses, oxidative stress, and tissue damage are described not only in overloaded skeletal muscle, but also in other internal organs. Furthermore, we introduce preventive countermeasures against the exhaustive exercise-induced pathogenesis together with the possibility of antioxidant interventions.

## 1. Introduction

Exercise-induced leukocytosis and delayed-onset muscle soreness are well-known phenomena. Redness, swelling, fever, pain, and loss of function are characteristic of inflammation, which is accompanied by leukocyte infiltration, oxidative stress, and production of pro-inflammatory cytokines. Indeed, there has been a tremendous accumulation of research on exercise-induced oxidative stress together with inflammation including from our own research group [1]. However, what are the interactions of oxidative stress and inflammatory mediator cytokines? Also, which are more important for the pathogenic role in exercise-induced tissue damage and as sensitive biomarkers for assessing exercise effects? These are two important questions whose answers remain unknown. Herein, in addition to basic background information on cytokines, research findings on exertional effects on cytokine release and the underlying mechanisms and triggers are introduced. Furthermore, the associations among cytokine responses, oxidative stress and tissue damage are described in overloaded skeletal muscle and other internal organs. Finally, we introduce potential preventive countermeasures against pathogenesis together with the possibility of antioxidant interventions without harmful side-effects.

## 2. Background Knowledge of Cytokine Function and Release

Cytokines are a diverse family of intercellular signaling molecules that regulate inflammation and immune responses [2,3]. Cytokines are produced by a variety of cells and usually act in an autocrine or paracrine manner at very low concentrations in tissues. Localized inflammation is usually a physiological protective response to initial tissue injury [3]. However, an elevated response can result in cytokine release into the circulation, which becomes pathogenic, self-destructive, and sometimes fatal to the host [2,3]. Systemic inflammatory response syndrome (SIRS) is a condition that is described by the resultant systemic cytokine release (hypercytokinemia, known as cytokine storm). The overproduction of proinflammatory cytokines leads to multiple organ damage, associated with numerous serious acute insults such as severe trauma, thermal injury, ischemia-reperfusion injury, septic shock, and systemic infections [3,4].

Tumor necrosis factor (TNF)-α, interleukin (IL)-1β, IL-6, IL-12, and IL-17 are elevated in sepsis. These pro-inflammatory cytokines are considered the main cytokines in pathogenesis of sepsis. TNF-α appears to be the first cytokine released systemically and peaks within several hours after the onset of sepsis, followed shortly thereafter by peaks in IL-1 and then IL-6 [5,6]. These proinflammatory cytokines induce pyrogenesis and promote subsequent acute inflammatory responses such as leukocytosis (neutrophilia) by inducing granulocyte colony-stimulating factor (G-CSF) and chemokines (abbreviated from chemotactic cytokines) such as IL-8 and monocyte chemotactic protein (MCP)-1 [1,2,5,6].

There are also compensatory anti-inflammatory responses to re-establish homeostasis; some anti-inflammatory cytokines—including IL-1 receptor antagonist (IL-1ra), IL-4 and IL-10—are released into the circulation and dampen the proinflammatory cytokine cascade [5,6]. IL-12 is classified as a major immunomodulatory cytokine, activating cellular immunity. Bioactive IL-12 p70 is a heterodimer composed of two subunits: p35 and p40. The IL-12 p40 homodimer in the absence of p35 expression and free p40 monomer does not mediate IL-12 activity, but acts as an IL-12 antagonist and shares the activity with IL-6 and IL-23, the other neutrophil activating cytokines [2,3,6,7]. As such, some complexities exist in the cytokine network in itself for regulation of inflammation.

## 3. Cytokine Kinetics in Response to Exercise

Exhaustive endurance exercise induces leukocytosis mainly due to neutrophilia in the systemic circulation, muscle and internal organ damage, and immune suppression [1,8,9,10,11,12,13]. To determine the underlying mechanisms of these phenomena, much attention has been focused on cytokines released into the circulation following exercise. Indeed, many studies have consistently shown that IL-1ra, IL-6, IL-8, and IL-10 increase markedly following endurance exercise lasting longer than several hours, such as marathons and triathlons [5,6,8,10,14,15,16,17,18,19,20]. However, the response of these cytokines is not so significant during and after short-duration intensive exercise [6,7,20,21,22,23,24,25,26,27] and eccentric-contraction exercise [28,29,30,31,32]. These responses are not dependent on exercise-induced muscle damage but are related to exercise intensity (physiological load/stress) [5,6,23].

Indeed, it has been demonstrated that IL-6 response to endurance exercise depends on decreased cellular energy levels and increased heat stress and subsequently correlates with stress hormone responses; however, they are suppressed by increased energy supply [5,8,10,33,34,35,36] and prior body-cooling interventions [4,33]. Furthermore, IL-6 enhances utilization of energy substrates such as free fatty acids, which contribute to endurance performance [5,6,27,35], whilst also inducing neutrophil mobilization and activation together with the anti-inflammatory cytokine release of IL-1ra and IL-10 [1,5,6,13,18,19,36]. Here, IL-1ra is a natural antagonistic cytokine that competes with IL-1 for receptor binding without inducing signal transduction, whilst IL-10 is the most immunosuppressive cytokine. Endurance exercise also increases plasma levels of IL-4 and IL-12p40 (IL-12 antagonist), which might work to block cellular immune response, cause susceptibility to infections [9,10,19,34,35], and might promote inflammation as a component of IL-6 and IL-23 (neutrophil activator).

Chemokines regulate tissue infiltration of leukocytes. IL-8 is a potent neutrophil chemotactic and activation protein referred to as neutrophil activating peptide 1 (NAP-1). IL-8 is released into the circulation under prolonged, intense exercise conditions [5,6,13,18,19,34], whereas short-time intensive exercise also enhances plasma IL-8 concentration [6,23,33]. These findings suggest that not only the duration but also the intensity of exercise might be important for IL-8 release. MCP-1 facilitates infiltration and activation of monocytes and macrophages. We demonstrated that MCP-1 concentration increased significantly not only in plasma but also urine following a marathon race and immediately after short-duration intensive exercise [6,18]. Also, IL-6 and G-CSF are involved in neutrophil mobilization from bone marrow reserves to the circulation after exercise [6,7,13]. Although neutrophils are involved in exercise-induced muscle damage and inflammation, we recently demonstrated that neutrophils mobilized into muscle contribute to exacerbating muscle injury by upregulating proinflammatory cytokine expression through the induction of macrophage infiltration with MCP-1 [37,38]. From this point of view, leukocytosis (neutrophilia) and related variables noted above can be good predictive indicators of exhaustive exercise-induced muscle and other organ damage/dysfunction [1].

## 4. Examples of Countermeasures to Exercise-Induced Inflammation

Although pre-exercise cooling can effectively attenuate systemic inflammatory response to exhaustive exercise [4,25,33,39,40], post-exercise cooling may not have significant effects [31,33]. Fluid intake has been reported to prevent systemic induction of IL-6 and neutrophil activation markers [41], whilst intensive exercise in the menstruation phase of the menstruation cycle increases systemic inflammation [26]. During the day, evening exercise induces more marked IL-6 release than morning exercise [27]. From a nutraceutical perspective, curcumin ingestion has reduced muscle inflammation after downhill running in a mouse model of exercise-induced muscle damage [42], but the evidence is not sufficient for other antioxidant and/or anti-inflammatory substances for the prevention of muscle damage and inflammation. Intestinal permeability increases following exercise, and endotoxemia occurs, which induces systemic inflammation [4,39,43,44]. Therefore, other countermeasures such as intake of some functional foods for gut barrier protection, bioavailability, and distribution [3,45,46,47,48,49,50,51,52], and appropriate immune responsiveness should be examined in future studies.

## 5. Conclusions and Future Directions

**Figure 1:**
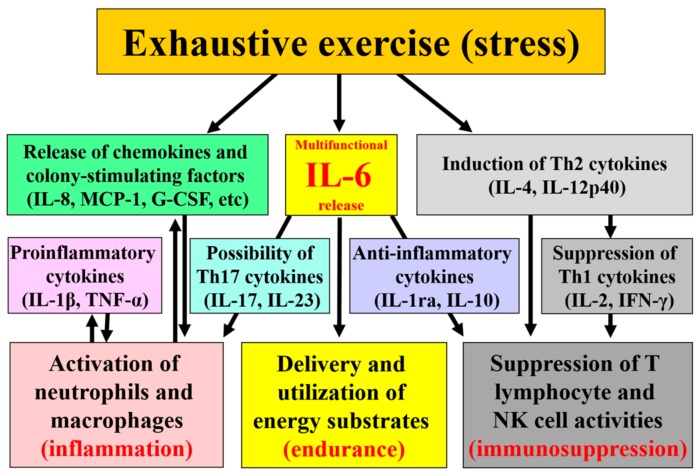
Cytokines mediate exercise-induced inflammation, immunosuppression and energy metabolism.

Whilst IL-6 might be good for athletes in optimizing energy substrate utilization for endurance performance on one hand, it may compromise the immune status of the athlete on the other hand by inducing systemic inflammation and increasing susceptibility to infections (Figure 1). Accumulation of inflammatory cytokines, neutrophils, and macrophages within organs are involved in tissue damage/dysfunction of not only muscles but also of the kidney, liver, and intestines which have recently been investigated in reference to the pathogenesis of multiple organ failure in heat stroke and sepsis and their underlying mechanisms. It is possible that appropriate countermeasures such as exercising in cool environments, and ingesting sufficient energy and fluids together with some functional food(s) might help to maintain endurance performance without causing harmful side effects on health. However, insight into both what does and does not work in regard to exercise-induced inflammation drawn from experimental studies is not enough at present and accumulation of research findings is necessary regardless of positive or negative results. Also, a future focus of countermeasures research is required regarding how the intestines and their permeability may be preserved by functional foods. These countermeasures may not only help to reduce heat illness and promote recovery, but also lead to the introduction of new research findings for the defense to stress and infection.
